# Patient perspectives of weight stigma across the cancer continuum: A scoping review

**DOI:** 10.1002/cam4.6882

**Published:** 2024-01-11

**Authors:** Nanako Hawley, Jennifer Green, Erica Ahlich, Caitlyn Hauff, Janice Hermer, Meghan B. Skiba, Dara L. James, Sarah H. Nash

**Affiliations:** ^1^ Department of Psychology University of South Alabama Mobile Alabama USA; ^2^ School of Exercise and Nutritional Sciences San Diego State University San Diego California USA; ^3^ Department of Health, Kinesiology, and Sport University of South Alabama Mobile Alabama USA; ^4^ Arizona State University Library Arizona State University Tempe Arizona USA; ^5^ College of Nursing University of Arizona Tucson Arizona USA; ^6^ College of Nursing University of South Alabama Mobile Alabama USA; ^7^ Edson College of Nursing and Health Innovation Arizona State University Tempe Arizona USA; ^8^ Department of Epidemiology University of Iowa Iowa Iowa USA; ^9^ Holden Comprehensive Cancer Center University of Iowa Iowa Iowa USA

**Keywords:** discrimination, health disparity, obesity, oncology, weight bias, weight stigma

## Abstract

**Background:**

Weight stigma has been defined as the social devaluation and denigration of individuals because of their weight. The purpose of this scoping systematic review was to assess and understand patient experiences with weight stigma in the cancer care setting.

**Method**s**:**

We conducted a systematic scoping review of studies examining shame, prejudice, bias, and stigma in relation to weight and cancer‐related care using five databases: PubMed, CINAHL Plus Full Text (ProQuest), Cochrane Library, PsycINFO (EBSCO), and Scopus. Articles were uploaded into Covidence for de‐duplication and screening. Included studies were peer reviewed, reported adult patient experiences in cancer‐related care, and were published in English between October 2012 and February 2023. Study characteristics and key findings were abstracted and qualitatively synthesized.

**Results:**

Publications meeting inclusion criteria yielded five studies (*n* = 113 participants). Most focused on the experiences of women (*n* = 4) and cancers which predominantly impact women (i.e., breast, cervical, endometrial; *n* = 4). All stages of the cancer continuum were included with studies examining screening (*n* = 2), treatment (*n* = 1), and post‐treatment survivorship (*n* = 2). Weight discrimination was discussed in four studies and weight‐biased stereotypes were discussed in three studies. Experiences of weight bias internalization were reported in four studies. One study described an instance of implicit weight bias.

**Conclusions:**

Limited studies examine patient experiences of weight stigma in cancer care; however, current evidence suggests that patients do experience weight stigma in cancer‐related care. This review highlights critical gaps and a need for more research on the prevalence and impact of weight stigma in cancer screening and care.

## 1 INTRODUCTION

Weight stigma is the social devaluation and denigration of individuals based on their body weight.^1^, ^2^ Weight stigma contributes to negative stereotypes (i.e., a generalized belief) about those with higher body weights and discrimination by others (i.e., negative action or unfair treatment toward individuals with overweight and/or obesity).^1^, ^3^, ^4^ Idealization of “thinness” in popular media has likely contributed to issues of weight stigma in the United States (U.S.).^5^, ^6^ For example, beginning in the 1980s and 1990s, the documented increase in the number of female models appearing notably thin in fashion magazines^7^, ^8^ was accompanied by a concurrent increase in exposure due to full body images becoming more popular on magazine covers during this same time period.^7^ This emphasis on thinness in the media has continued to the present day; however, newer research suggests popular media images of both men and women have become even more difficult and unrealistic for most people, emphasizing both thinness and muscularity (i.e., the so‐called “fit” ideal^9^, ^10^, ^11^).

Data from the National Survey of Midlife Development suggested weight discrimination is among the most common types of discrimination reported by U.S. adults.^12^ Research conducted within the past decade reveals that weight stigma has increased globally,^13^ despite decreases in other forms of discrimination (i.e., sexual orientation and race).^14^ Pooled estimates from a systematic review and meta‐analysis found prevalence of perceived weight discrimination ranged from 19.2% to 41.8% among individuals with a body mass index (BMI) between 30 and 35 kg/m^2^ and above 35 kg/m^2^, respectively.^15^ Weight discrimination has been documented across the most fundamental sectors of society, including employment, education, and health care.^16^, ^17^

The prevalence and impact of weight stigma in healthcare settings is well documented.^12^, ^18^, ^19^ Puhl and Brownell^20^ found that 69% of individuals with obesity felt stigmatized by their physicians. Substantial evidence suggests that primary care providers report less respect for patients with obesity^21^, ^22^ and spend less time educating patients with obesity about their own health, resulting in low quality interactions with providers.^4^, ^23^, ^24^ Further, health care professionals tend to give unsolicited weight‐related treatment recommendations to heavier patients, even when patients are not seeking treatment for a concern related to their weight.^25^ Even among health professionals specializing in obesity, significant implicit (i.e., subconscious negative attitudes and stereotypes) and explicit (i.e., overt, consciously held negative attitudes) anti‐fat biases exist, including the stereotypes that people with obesity are lazy, stupid, and worthless.^26^ Experiences of weight stigma faced by patients with obesity are associated with several negative behavioral and psychological health outcomes, including binge eating, reduced motivation for and engagement in physical activity, lower self‐esteem, and poorer health‐related quality of life and overall worse mental health.^27^, ^28^, ^29^, ^30^

Compounding the negative impacts of weight stigma are one's own self‐directed biases. More than half of adults with overweight and/or obesity in the U.S. report high levels of weight bias internalization.^31^ Weight bias internalization occurs when individuals engage in self‐blame and self‐directed weight stigma because of their weight, and often includes agreement with stereotypes and application of these stereotypes to oneself.^2^ A recent systematic review found that weight bias internalization is a significant predictor of depression, anxiety, poor body image, disordered eating, and low quality of life.^32^ Research has also demonstrated associations between weight bias internalization and lower physical activity; however, additional studies with increased methodological rigor are required to further understand this relationship.^12^

Importantly, weight stigma (experienced externally or internalized) is a leading contributor to healthcare avoidance, a type of patient disengagement that impedes an individual's health seeking behaviors and may cause them to delay seeking healthcare.^12^, ^21^, ^33^ Healthcare avoidance may further exacerbate existing comorbidities among those living with obesity, and increase risk of developing new comorbidities, including cancer. For example, women with obesity are less likely to be recommended for Pap testing for cervical cancer than those without obesity and are less likely to receive screening.^34^ This may have serious implications: women with obesity are also less likely to be diagnosed with cervical precancers, and more likely to be diagnosed with cervical malignancies.^35^ In addition, despite obesity being a risk factor for postmenopausal breast cancer, women with obesity are less likely to receive either mammography^36^ or clinical breast exams,^37^ or MRI,^38^ and are more likely to be diagnosed with breast cancer at later stages. Lack of access to cancer screening, engaging in healthcare avoidance, and/or weight/body size limitations of medical equipment (e.g., MRI, gowns, etc.) can potentially delay treatment initiation for cancer among individuals with overweight and/or obesity^39^ and are associated with negative cancer outcomes.^38^ Such delays have serious implications: cancer survival is lower at later stage at diagnosis for almost all cancer sites, and treatment delay is associated with a 6%–23% increased mortality risk.^40^ Finally, weight stigma may exacerbate existing cancer disparities. Those who experience health disparities related to racial and ethnic identity, socioeconomic status, and nativity in the U.S., and who are also living with overweight and/or obesity, are significantly less likely to utilize preventive health care services.^41^

While weight stigma is a common occurrence in the healthcare setting, there is a lack of understanding about experiences of weight stigma more specifically during cancer care. A review of the literature is critically needed to synthesize the growing body of literature documenting experiences of weight stigma specifically in cancer care. The purpose of the current scoping review was to explore patient experiences of weight stigma across cancer care settings and map operational definitions of weight stigma and related constructs on the existing evidence. Findings from this review will serve as a call to action for additional research in this area, and this review will provide specific recommendations for future directions.

## 2 METHODS

This systematic scoping review was conducted following the Preferred Reporting Items for Systematic Reviews and Meta‐Analyses checklist extension for Scoping Reviews (PRISMA‐ScR).^42^ The review protocol was registered at Open Science Framework, Registration DOI: https://doi.org/10.17605/OSF.IO/F8EV2.

### 2.1 Eligibility criteria

The population, concept, and context (PCC) framework guided the inclusion of studies in this review.^43^ The population (P) of interest included all people with overweight or obesity who had experienced weight stigma in the cancer care setting; weight stigma was the concept (C) underlying this work, and the context (C) referred to cancer care across the continuum, from screening, through diagnosis, treatment, and survivorship care. In this review, weight stigma and related concepts were guided by definitions established by the Joint International Consensus Statement for Ending Stigma of obesity (Box 1).^2^ To meet inclusion criteria, articles must have been peer reviewed, focused on adult patients being screened, treated, or followed for cancer, and reported patient experiences in cancer‐related care. Articles that were not published in English, included children, not cancer care specific, or focused on the perspectives of providers and/or provider education were excluded.

BOX 1 **Definitions of weight stigma used to guide this review, taken directly from the Joint International Consensus Statement for Ending the stigma of obesity (2).**

Weight stigma refers to social devaluation and denigration of individuals because of their excess body weight, and can lead to negative attitudes, stereotypes, prejudice, and discrimination.

Weight‐based stereotypes include generalizations that individuals with overweight or obesity are lazy, gluttonous, lacking in willpower and self‐discipline, incompetent, unmotivated to improve their health, non‐compliant with medical treatment, and are personally to blame for their higher body weight.

Weight discrimination refers to overt forms of weight‐based prejudice and unfair treatment (biased behaviors) toward individuals with overweight or obesity.

Weight bias internalization occurs when individuals engage in self‐blame and self‐directed weight stigma because of their weight. Internalization includes agreement with stereotypes and application of these stereotypes to oneself and self‐devaluation.

Explicit weight bias refers to overt, consciously held negative attitudes that can be measured by self‐report.

Implicit weight bias consists of automatic, negative attributions and stereotypes existing outside of conscious awareness.

### 2.2 Information sources and search strategy

Comprehensive literature searches for article were completed on January 11, 2023 by a university‐associated Health Science librarian (J.H.). No date constrictions were placed on the search. The databases searched included PubMed, CINAHL Plus Full Text (ProQuest), Cochrane Library, PsycINFO (EBSCO), and Scopus, with results uploaded into Covidence, a web‐based program designed to manage systematic reviews^44^ for de‐duplication and screening. During initial screening, it was discovered that some aspects of the topic had not been fully articulated; subsequently, an updated search was run on February 21, 2023, with results uploaded to Covidence for de‐duplication and screening. The searches were optimized for each individual database but included a combination of keywords and subject headings for the following three categories: weight/body image; shame/prejudice/stigma/bias; cancer (see Appendix A for full search strategies).

In the first stage of screening, article titles and abstracts were independently evaluated for relevancy by three authors (N.A.H., S.H.N, & J.G). Disagreements in title and abstract screening were independently evaluated by one author (D.L.J). During the second stage of screening, three authors (N.A.H., S.H.N, & J.G.) conducted a full text screening to evaluate if articles were eligible and reflective of inclusion criteria. Discrepancies in full text screening were independently evaluated by one author (D.L.J), and discussed by all authors until consensus was reached. Qualitative interpretation was conducted from included segments of transcripts; full transcripts were not available for review.

### 2.3 Quality assessment and data extraction

Studies were independently assessed for quality using the Critical Appraisal Skills Programme (CASP)^45^ checklist for qualitative studies by three authors (E.A., J.G., & N.A.H), with any disputes discussed by all authors until consensus was reached. The CASP qualitative research checklist includes five questions: (1) Was there a clear statement of aims of the research? (2) Is a qualitative methodology appropriate? (3) Was the research design appropriate to address the aims of the research? (4) Was the recruitment strategy appropriate for the aims of the research, and (5) Was the data collected in such a way that addressed the research issue? Reported results were coded for categories of weight related experiences (i.e., weight stigma, weight‐based stereotypes, weight discrimination, weight bias internalization, explicit weight bias and implicit weight bias). Categorization was followed definitions previously established, and given in Box 1.^2^

## 3 RESULTS

### 3.1 Literature search and study selection

**FIGURE 1:**
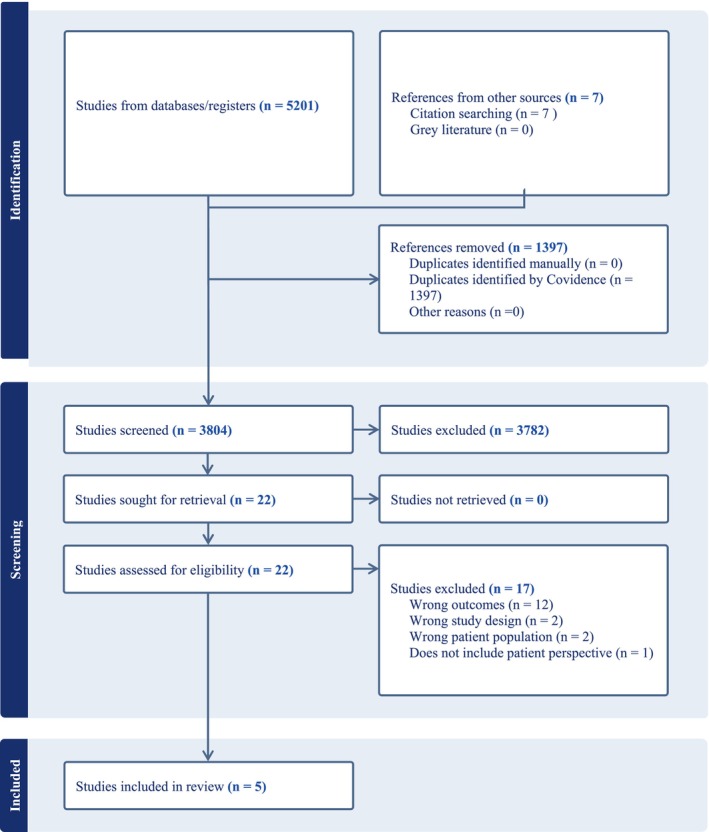
PRISMA CONSORT diagram for this systematic scoping review.

Figure 1 gives the PRISMA CONSORT diagram for this systematic scoping review. There were 5201 articles identified from literature searches, and seven from hand searching, for a total of 5208 articles. Of these, 1397 duplicates were removed, leaving 3804 studies for screening. In the first stage, 3782 studies were excluded, leaving 22 articles advancing to full text review. After the full text review, 17 more studies were excluded (e.g., wrong outcomes, study design, patient population, or did not include patient perspective), leaving five studies for quality assessment evaluation (See Figure 1).

### 3.2 Study characteristics

Characteristics of the studies included in this review are given in Table 1. All five studies included used a qualitative research design, all studies met all five quality criteria from the CASP qualitative research checklist. Of the five studies included, two were conducted in the U.S.,^46^, ^47^ two were conducted in Canada,^48^, ^49^ and one was conducted in Australia.^50^ Sample sizes ranged from 11 to 15 participants per study with a total of 113 participants overall. The majority of studies focused on the experiences of women only^47^, ^48^, ^49^, ^50^ while one study included both women and men.^46^ All studies included adult participants with mean ages ranging from 55.5 to 65.3 years. Study samples were predominately overweight or obese based on BMI criteria. Cancer types were predominantly those which impact women, including breast cancer (*n* = 3),^47^, ^49^, ^50^ cervical cancer (*n* = 1),^47^ endometrial cancer (*n* = 1),^48^ and one study with heterogeneous cancer diagnoses (31% prostate).^46^ All stages of the cancer continuum were represented, with two studies examining care during cancer screening,^47^, ^50^ one study focusing on cancer treatment,^48^ and two studies focusing on post‐treatment survivorship.^46^, ^49^

**TABLE 1:** Characteristics of the studies included in this scoping review (*n* = 5)^a^.

Author (Year)	Country	Sample size	Sex	Mean age (y)	BMI	Race	Type of cancer	Cancer stage
Beehler, 2014	USA	35	Female 6%, Male 94%	64	Normal 20%, Overweight 51%, Obesity 29%	White 80%, Non‐White 20%	Mixed	Survivorship
Cusimano, 2019	Canada	15	Female 100%	61	BMI >40 kg/m^2^	not provided	Endometrial	Treatment
Friedman, 2012	USA	33	Female 100%	55.8	not provided	White 51%, Black 45%, Mixed 3%	Breast & Cervical	Screening
McBride, 2019	Australia	19	Female 100%	57	BMI >30 kg/m^2^	not provided	Breast	Screening
Pila, 2018	Canada	11	Female 100%	65.31	not provided	White, 72.7%	Breast	Survivorship

^a^ Characteristics of study participants given as reported by authors.

### 3.3 Mapping weight stigma and related constructs

Operational definitions of weight stigma and related constructs were either absent in the studies reviewed, or terminology used inconsistently between studies. Additionally, studies did not themselves differentiate between the different constructs of weight stigma (e.g., weight‐based stereotypes, weight discrimination, weight bias internalization, explicit weight bias, and implicit weight bias). Therefore, in Table 2, we present findings from each of the five studies, mapped by our group to these different weight stigma‐related concepts.^2^ Experiences of weight stigma were detailed in all studies; this often took the form of weight‐based discrimination and weight‐based stereotyping, but weight bias internalization was also reported.

**TABLE 2:** Key findings of weight‐stigma and related constructs identified from the studies included in this review (*n* = 5).

Author (Year)	Terms	Supporting quotations	Examples	Key findings
Beehler 2014	Weight discrimination	“Like one provider I had when we changed, she said, ‘I want you to lose ten pounds by the next time you're in here, really seriously, or I'm going to be upset with you.’ I lost ten pounds. I busted my butt and I just did it for her maybe in the wrong way but it wasn't probably a healthy way of losing weight. It was more of almost starvation to lose the weight so she wouldn't be mad at me”.	Being “commanded” to lose weight in an unrealistic fashion by a healthcare provider.	There are many barriers and facilitators (health service delivery factors) that exist in diverse domains for cancer survivors with veteran status, each of which offers unique challenges and opportunities for improving engagement in behavior change following cancer diagnosis and treatment. Healthcare providers who made unrealistic demands on cancer survivors with veteran status or who were punitive when goals were not achieved presented barriers to change.
Cusimano 2019	Weight discrimination	“She didn't like to deal with me because of my body weight. That's pretty much how I ended up in Toronto.”	Providers treat patients with obesity differently such as not receiving options for non‐curative alternatives to surgery because of their body weight. Providers judge or blame patients for cancer. Patients feel shame and self‐blame that their weight caused cancer. Patients feel shame and embarrassment for diagnostic/treatment procedures. Patients avoid healthcare due to prior negative experiences related to their weight. Barriers to care were obesity specific (e.g., difficulty obtaining biopsy/imaging, size/structure of waiting rooms, CT scanners, tables, and medical equipment), treatment delays (e.g., canceled appointments because patient was “high risk” and referring out, multiple referrals before finding accepting surgical team).	Thematic analysis identified that patients with morbid obesity are subject to stigma and poor communication in the healthcare system. Although clinical, administrative, financial, geographic, and facility‐related barriers exist, quality care for patients with morbid obesity is an achievable goal.
	Weight‐based stereotypes	“When I've been thin, my treatment has differed vastly. Absolutely. I feel the gynecologist absolutely judged me. She totally berated me for my weight. Totally made it my fault that I had this illness, and the subtext was, you know, you brought this on yourself, and we'll try to help you as best you can… as best we can, but, you know, it's your doing.”		
	Weight bias internalization	“It's made me very angry and disgusted with myself. Cause, you know, it's like I have brought this problem on myself, even though I know that people who are not obese could still get this.”		
Friedman 2012	Weight‐based stereotypes	“I think they do‐what would be the expression…put you in a box. ‘Oh, you're just lazy or you just don't care. You're not doing what you need to do, so you're not going to do what I tell you to do anyway’.”	Negative interactions with physicians or staff (insensitive comments). Doctors blame all health problems of women with obesity on their weight or always focus on weight. Equipment and gowns could not accommodate women with obesity. Assuming women with obesity cannot do certain actions (e.g., climb onto exam table). Women with obesity feeling weight‐related shame.	Participants verified many barriers to cervical and breast cancer screening previously identified in the general population and highlighted several weight‐related barriers. Participants who followed through on their cancer screenings may share certain personality traits, such as conscientiousness or self‐regulatory ability, that allow them to complete difficult or feared tasks. Personality may act as an important mediator in health behavior and should be taken into account in future theoretical models and health behavior interventions, particularly for obese women.
	Weight discrimination	“I always have to ask for a bigger gown, and that's embarrassing. Especially when somebody else is in the stall. They always forget to put one or two big gowns”.		
	Weight bias internalization	“I was going to this one doctor, and it was a bad experience because I was overweight and he like made it a point like…actually, I didn't even go back to him because he was like, ‘You've got to lose weight. You've got to lose weight. You're too heavy,’ but it was the way he was saying it. I mean it was the truth, he wasn't lying, but it was just the way he said it to me, and I felt like if I wasn't overweight, I wouldn't have been treated that way”.		
McBride 2019	Weight discrimination	“Yeah, a person handling, manhandling your breasts in to place…not every practitioner that I've met, not every mammographic that I've met had that, had that sensitivity…one of them…I actually thought about making a complaint, and then I thought look, there's not really any complaint that I can make. He needs to get my breast on to the plate. You know, the fact that he's just acting like a bit of a pig is off but it's not enough to actually…you know, to put a complaint in anywhere. But certainly, you know, the sensitivity for what it might be like for women who don't feel…like I say, women who don't feel okay about their body”.	Pain related to having larger breasts. Mistreatment during procedure. Feelings of self‐consciousness and body image concerns contributed to negative experiences and perceptions of screening which led to increased reluctance or avoidance of the procedure. The sensitivity of the radiographer was important in how body image concerns were managed (lack of sensitivity and communication about the procedures). Increased anxiety due to multiple images needed (thinking something was wrong). Patients agree with an apply stereotypes to themselves.	In patients, low knowledge around a heightened need to screen existed, they also reported limited desire to prioritize personal health needs, reluctance to screen due to poor body image and prior negative mammographic experiences due to issues with weight.
	Weight bias internalization	“No, no, not really. I think…no. I think that you…in my head I just accept the fact that you know, I'm one of the fat ones, that's all, but then I give them a lot more to work with, so they're really pushed to the edge. I make sure they can do it properly. Anyone can get a little boob in there. You try and get this big one in.”		
Pila 2018	Weight‐based stereotypes	“I asked her last November, ‘is there risk of me relapsing as I grow older?’ and she said you should be ok as long as you don't get fat, that's what she told me.”	Women reported weight‐related distress, shame, blame, and embarrassment. Women desire more support from doctors to recommend weight loss. Providers made Insensitive comments. Experience at doctor's offices are stressful around the issue of weight. Women have self‐presentational desires at doctor's appointments (e.g., appointment used as temporal target to achieve weight loss goal to satisfy doctor).	Five themes were identified: weight concerns contributed to psychological distress, prevalent history of weight cycling and ongoing quest to manage weight, shifting psychological impact of cancer versus weight, perceptions of failure around goal‐oriented weight management behaviors, and internalized and explicit social pressures for weight loss in the context of risk reduction. In light of the fundamental challenges of weight management, and the present findings, improving weight‐related distress should be a clinical priority to improve the well‐being of women in survivorship.
	Weight bias internalization	“I am always scared and embarrassed to get on the scale at the doctor's office… I even take a laxative a couple of days before my appointment to decrease my weight. I also weigh the clothes I am wearing to the doctors and choose the lightest ones. I also wear a body shaper to hide my rolls”.		

### 3.4 Cancer care experiences and weight discrimination

Four studies discussed weight discrimination experienced during cancer care.^46^, ^47^, ^48^, ^50^ While the studies did not explicitly define weight discrimination, we identified this when study participants mentioned being treated differently because of their weight. Discriminatory behaviors included not receiving options for non‐curative alternatives to surgery because of their body weight,^48^ treatment delays,^48^ mistreatment during the procedure,^50^ and not having appropriate medical equipment to accommodate a larger body size (e.g., gowns, tables, CT scanners, waiting rooms).^47^, ^48^

### 3.5 Cancer care experiences and weight‐based stereotypes

While no studies explicitly defined weight‐based stereotypes, we were able to map instances of stereotyping based on accounts from participants in three studies.^47^, ^48^, ^49^ Participants mentioned feeling judged or blamed by providers for their higher body weight and/or illness.^46^, ^47^, ^48^ Participants in other studies described insensitive comments from providers using hurtful terms such as “lazy”^47^ or “fat”.^49^ Other instances of stereotyping included providers telling patients that they were not doing what they need to be doing (for their health) or assuming they would not do anything they are told in the future.^47^

### 3.6 Cancer care experiences and weight bias internalization

Four of the five articles described instances of weight bias internalization.^47^, ^48^, ^49^, ^50^ Participants mentioned feeling shame and embarrassment about their weight^47^, ^48^, ^49^, ^50^ and about certain diagnostic/treatment procedures because of their weight.^48^, ^50^ Some patients expressed that shame and embarrassment led to avoidance of medical procedures,^48^, ^50^ or engaging in behaviors designed to satisfy doctors at appointments (e.g., taking laxatives to present at lower body weight, wearing light clothing or body shapers).^49^ Other patient accounts mentioned feeling “angry” or “disgusted” with themselves^48^ or having increased anxiety or stress related to procedures or going the doctor's office.^49^, ^50^

## 4 DISCUSSION

This systematic scoping review identified five qualitative studies that reported patient experiences of weight‐based stigma across the cancer continuum, highlighting a dearth of research on this topic. Several instances of weight‐based discrimination or stereotyping experienced by patients were identified. Discriminatory behaviors were experienced across the cancer continuum and included not being offered non‐curative therapies because of their body weight, treatment delays, mistreatment by clinicians, and lack of appropriate medical equipment to accommodate a larger body size. Studies also reported patient experiences of weight‐based stereotyping by medical providers, and internalization of weight bias that led to shame and healthcare avoidance. Our findings indicate substantial gaps and areas for future research that are needed to comprehensively address the impacts of weight discrimination on patients with cancer and their cancer outcomes.

Well‐established definitions of weight stigma‐related concepts need to be consistently applied in research. We observed inconsistent use and definitions of weight‐based stigma and discrimination across the studies included in the current review; yet, we were able to extract reports of patient experiences that matched the definitions used herein. Standardization of terminology is critical to facilitate high quality research, comparability across studies, and participant understanding of central concepts. The use of consistent definitions is particularly important where studies examine different facets of weight stigma simultaneously. For example, when both experiences of discrimination and weight bias internalization are included, the explicit definition of these concepts ensure everyone is clear on any differences between these concepts and how they are used. The recent Joint International Consensus Statement for Ending Stigma of Obesity statement by Rubino and colleagues^2^ employed in this review provides clear and concise definitions researchers may consider using. Additionally, standardized and respectful language used in research and practice can assist in overcoming biases.^51^ Use of person‐first and patient‐first language in overweight and obesity clinical practice better demonstrates respect, non‐stigmatizing communication, and reduces harm.^2^ Increasing trends toward the use of person‐first language in the obesity scientific literature since 2004 has reduced weight stigma in research.^52^ Translation of person‐first language into clinical practice presents both additional benefits as well as challenges.

Further, while the studies represented in the current review spanned the cancer continuum from screening to post‐treatment survivorship, there is a lack of research explicitly comparing prevalence and experiences of weight stigma as an individual navigates cancer care across the continuum and the impact of these experiences on cancer outcomes. While there is a growing body of evidence to indicate that most individuals with obesity have experienced weight stigma, most of this research has been focused on everyday life.^53^ When studies have focused on experiences within healthcare, it has primarily been in the primary care setting.^12^ On the provider side, there is clear evidence to suggest that most primary care doctors and medical trainees report either explicit or implicit bias against persons with obesity^18^, ^54^; yet, to our knowledge, no evidence exists to understand anti‐fat bias exhibited by cancer treatment providers. Identifying how provider anti‐fat bias and patient experiences of weight stigma vary across the continuum will inform the design of interventions to reduce patient experiences of weight stigma, improving both patient experience and outcomes.

To date, no interventions have focused specifically on addressing weight stigma experienced by patients with cancer; however, several approaches have been explored in other settings that could be explored with this patient population. For example, a systematic review of Health at Every Size® (HAES) interventions for individuals showed positive influence on cardiometabolic measures of health as well as improved overall well‐being and improved body image, but not the impact of stigma.^55^ A randomized controlled trial of a program providing psychoeducation on weight management and weight stigma showed improvements in weight self‐stigma compared to those receiving only weight management education.^56^ Teaching patients self‐compassion may also moderate weight bias internalization among individuals with overweight and/or obesity.^57^ Unfortunately, most interventions addressing weight stigma have focused on the patient, rather than the organizational or systemic structures. Shifting focus from patients to providers to reduce anti‐fat bias may is one option to reduce experiences of weight stigma in the clinical setting.^58^ The Joint International Consensus Statement identified five recommendations for current health care professionals to provide more compassionate health care for individuals with overweight and/or obesity: (1) see, acknowledge, and treat the whole person; (2) identify bias and assumptions; (3) practice patient‐centered communication; (4) create a welcoming environment; and (5) pursue lifelong learning.^59^ Randomized controlled trials to understand the most effective ways to reduce weight stigma in oncology, including multi‐level interventions that address bias at the provider and organizational levels, are sorely needed.

### 4.1 Strengths and limitations

This scoping review adds to the existing knowledge of weight stigma in healthcare with specific focus on the cancer continuum. The strength of this scoping review included rigorous methods to identify observational quantitative and qualitative studies and assess quality. Consistent themes related to the experience of weight stigma concepts and cancer care were identified using a previously published consensus definition.^2^

Our findings may be limited by the variability in definitions of weight stigma and related concepts used in published studies, meaning our search may have missed some published articles. This was in part addressed by hand‐screening bibliographies of included articles identified by search algorithms. Our search terms referred to the biases experienced by individuals with overweight and/or obesity, not underweight. As such, the focus of this scoping review is on overweight and/or obesity and does not encompass the full spectrum of body weights and sizes. Individuals with very low body weights may also experience weight‐bias^60^; these experiences were not captured in this review. In our included studies, overweight and/or obesity were defined using BMI and previously established cut points; yet, BMI acts only as a surrogate measure of fatness, and has often incorrectly been used as an indicator of health.^61^ Moreover, studies were conducted in a small number of Western nations (U.S., Canada, Australia), potentially limiting the generalizability of findings, particularly where health systems differ substantially. While is possible that experiences of weight stigma vary across countries, studies in the general healthcare setting indicate remarkable consistency across six countries, including those represented in this review.^12^ Finally, the included studies focused primarily on the experiences of women, and samples were older and mostly white, minimizing the generalizability of these findings to men, young adults, and marginalized populations. As described above, quantitative research using more representative samples is required to explore this issue further.

## 5 CONCLUSION

The purpose of this scoping systematic review was to explore patient experiences of weight stigma during their cancer care as well as map operational definitions of weight stigma and related constructs on the existing evidence. Though limited in number, available studies consistently suggest that patients experience weight stigma across all stages of cancer‐related care, including screening, treatment, and survivorship. When categorized based on definitions provided in the recent Joint International Consensus Statement for ending Stigma of Obesity,^2^ patient experiences ranged from stereotyping to discrimination to internalized weight bias. Several recommendations arise from our findings. First, well‐established definitions of weight stigma‐related concepts need to be consistently applied in research. Second, additional research is needed to quantify prevalence of weight stigma among patients with cancer; to compare experiences of weight stigma across the cancer continuum; to understand impacts of weight stigma on cancer treatment and outcomes; and to understand anti‐fat bias exhibited by cancer treatment providers. Finally, once weight stigma experienced by patients with has been comprehensively documented, multi‐level interventions to address anti‐fat bias in the cancer treatment setting may be necessary. Given the prevalence of overweight and/or obesity across the globe, it is critical that research drives the science forward to understand and directly address the prevalence and effect of weight stigma across the cancer continuum.

## AUTHOR CONTRIBUTIONS

**Nanako Hawley:** Conceptualization (equal); formal analysis (equal); investigation (equal); methodology (equal); project administration (lead); writing – original draft (equal); writing – review and editing (equal). **Jennifer Green:** Conceptualization (equal); formal analysis (equal); investigation (equal); methodology (equal); writing – original draft (equal); writing – review and editing (equal). **Erica Ahlich:** Conceptualization (equal); formal analysis (equal); investigation (equal); methodology (equal); writing – original draft (equal); writing – review and editing (equal). **Caitlyn Hauff:** Conceptualization (equal); formal analysis (equal); investigation (equal); methodology (equal); writing – original draft (equal); writing – review and editing (equal). **Janice Hermer:** Data curation (equal); formal analysis (equal); investigation (equal); methodology (equal); writing – review and editing (equal). **Meghan B. Skiba:** Conceptualization (equal); formal analysis (equal); investigation (equal); methodology (equal); writing – original draft (equal); writing – review and editing (equal). **Dara L. James:** Conceptualization (equal); formal analysis (equal); investigation (equal); methodology (equal); supervision (equal); writing – original draft (equal); writing – review and editing (equal). **Sarah Nash:** Conceptualization (equal); formal analysis (equal); investigation (equal); methodology (equal); supervision (equal); writing – original draft (equal); writing – review and editing (equal).

## FUNDING INFORMATION

Research reported in the manuscript was supported, in part by the National Center for Advancing Translational Sciences of the National Institutes of Health under award number KL2TR003097 for Dr. James. Dr. Nash is supported by P30 CA086862 and an Early Career Scholar Award from the University of Iowa Vice President for Research. Drs. Skiba, James and Nash were participants in the 2022 Transdisciplinary Research in Energetics and Cancer Workshop (R25CA203650). The content is solely the responsibility of the authors and does not necessarily represent the official views of the National Institutes of Health.

## CONFLICT OF INTEREST STATEMENT

The authors do not have any conflicts of interest to disclose.

## Supporting information


Appendix A.

## Data Availability

All data included in this systematic scoping review have been previously published.
