# Supplementary material for: Patient perspectives of weight stigma across the cancer continuum: A scoping review
Source: Cancer Med. 2024 Jan 11;13(3):e6882. doi: 10.1002/cam4.6882 (PMC10905240; doi:10.1002/cam4.6882)
Supplement: Supplementary file 1 — Appendix A. [file CAM4-13-e6882-s001.docx]

**Literature Searches**

**PsycInfo** 2023/01/25 = 75 results

(weight OR fat OR obese OR obesity OR overweight) AND (shame OR shaming OR bias OR stigma OR prejudice OR stigma OR discrimination OR discriminatory OR stereotype OR stereotyping ) AND cancer AND (female OR women OR woman)

Filters: Peer Reviewed, English, Adulthood (18 yrs & older)

**PubMed** 2023/01/25 = 2472 results

((((weight OR fat OR obesity OR obese OR overweight) OR (overweight[MeSH Terms])) OR (obesity[MeSH Terms])) OR (body weight[MeSH Terms])) AND ((shame OR shaming OR bias* OR stigma OR prejudice* OR discrimination OR discriminatory OR stereotype OR stereotyping) OR (weight prejudice[MeSH Terms])) AND (cancer OR neoplasms)

Filters: Female, Adult 19+ years, English

**CINAHL Plus Full Text** 2023/01/75 =  93

( ( (MH "Obesity") OR (MH "body weight") ) OR ( overweight OR obese OR obesity OR fat ) ) AND ( (MH "Weight Bias") OR ( shame OR shaming OR bias* OR stigma OR prejudice* OR discrimination OR discriminatory OR stereotype OR stereotyping ) ) AND cancer

Filters: Peer Reviewed, English, All Adult, Female

**Cochrane Library** 2023/01/25= 36 Cochrane Reviews

(obese OR obesity OR overweight OR fat OR weight) AND (shame OR shaming OR bias OR stigma OR prejudice OR discrimination OR discriminatory OR stereotype) AND cancer AND (female OR women OR woman)

No Filters

**Scopus** 2023/01/25 = 945

( TITLE-ABS-KEY ( obese  OR  obesity  OR  overweight  OR  fat  OR  weight )  AND  TITLE-ABS-KEY ( shame  OR  shaming  OR  bias  OR  stigma  OR  prejudice  OR  discrimination  OR  discriminatory  OR  stereotype )  AND  TITLE-ABS-KEY ( cancer )  AND  TITLE-ABS-KEY ( female  OR  women  OR  woman )  AND  TITLE-ABS-KEY ( adult ) )

Filter: English

**Final Searches**

**PubMed** 2023/02/21  = 2,097 results

English only, All adults (19+) filters applied = 1072 results

((((((fat OR obesity OR obese OR overweight OR underweight OR "body composition" OR "body shape" OR "body mass index") OR (Obesity[MeSH Terms])) OR (Overweight[MeSH Terms])) OR (Thinness[MeSH Terms])) AND (((stigma OR bias* OR prejudice* OR discrimination OR discriminatory OR stereotyping) OR ("body dysmorphia" OR perception OR stigmatization OR Shame Or shaming OR "body surveillance" OR "weight surveillance")) OR (weight prejudice[MeSH Terms]) OR body dissatisfaction[MeSH] OR body dysmorphic disorders[MeSH])) AND (cancer[Title/Abstract] OR neoplasms[Title/Abstract])) NOT ("weighted imaging")

**CINAHL Plus Full Text (EBSCO)** 2023/02/21 = 810 results

English only, All Adult, & Academic Journals filters = 426 results

(MH "Thinness") OR MH Obesity OR ( fat OR obesity OR obese OR overweight OR underweight OR "body composition" OR "body shape" OR "body mass index" )

AND

(MH "Weight Bias") OR ( shame OR shaming OR bias* OR prejudice* OR discrimination OR discriminatory OR stereotyping ) OR ( "body image" OR "body dysmorphia" OR "body attitude" OR perception OR stigmatization OR "body surveillance" OR "weight surveillance" OR "weight prejudice" OR "body dissatisfaction" OR "body dysmorphic disorders" )

AND

TI ( Cancer OR oncology OR neoplasms ) OR AB ( Cancer OR oncology OR neoplasms )

NOT

“weighted imaging”

**PsycINFO (Proquest)** 2023/02/21 = 71 results

English, Adulthood (18 and older) & Scholarly journals filters = 36 results

(MAINSUBJECT.EXACT("Obesity") OR MAINSUBJECT.EXACT("Overweight") OR MAINSUBJECT.EXACT("Underweight") AND (fat OR obesity OR obese OR overweight OR underweight OR "body composition" OR "body shape" OR "body mass index" OR thinness)) AND ((MAINSUBJECT.EXACT("Obesity (Attitudes Toward)") OR MAINSUBJECT.EXACT("Obesity (Attitudes Toward)")) OR ((stigma OR bias* OR prejudice* OR discrimination OR discriminatory OR stereotyping) OR ("body dysmorphia" OR perception OR stigmatization OR Shame Or shaming OR "body surveillance" OR "weight surveillance") OR ("weight prejudice") OR "body dissatisfaction" OR "body dysmorphic disorders")) AND (MAINSUBJECT.EXACT("Neoplasms") OR tiab(cancer))

**Cochrane Library** 2023/02/21 = 38 Cochrane Reviews

obese OR obesity OR overweight OR fat OR underweight OR thinness in Title Abstract Keyword AND shame OR shaming OR bias OR prejudice OR discrimination OR discriminatory OR stereotype OR stigma OR dysmorphia OR "body image" in Title Abstract Keyword AND cancer OR neoplasms in Title Abstract Keyword

weight bias internalization - adding it made no difference to PubMed or PsycINFO searches on March 1, 2023
